# Supplementary material for: Spatial and temporal variations of aridity shape dung beetle assemblages towards the Sahara desert
Source: PeerJ. 2018 Sep 20;6:e5210. doi: 10.7717/peerj.5210 (PMC6151256; doi:10.7717/peerj.5210)
Supplement: Supplemental Information 1 — Table S1. Summary of grouped variables used (variables used in the final analyses have been marked with a tick mark ✓). Table S2. Localization (municipality and coordinates), altitude and amount of dung of each replicate and site sampled. Dung availability variables (cow dung and other dung) are presented as an average of the amount found in the four sampling campaigns. Aridity is here presented as the raw original variable, but note that for analysis the inverse of aridity was used in order to have directly interpretable results with increasing aridity. [file peerj-06-5210-s001.pdf]

Supplementary S1, Table 1.

Group	Variable-name	Used	Unit	Scale	Description	Source
**Vegetation**						
	Vegetation height		cm	Replicate (averaged to site/campaign)	Visually estimated vegetation height over an area of ca. 1000 m^2	Present sampling
	Vegetation cover		%	Replicate (averaged to site/campaign)	Visually estimated vegetation cover over an area of ca. 1000 m^2	Present sampling
**Dung**						
	Total dung	✓	g/100 m^2	Replicate (averaged to site/campaign)	Estimated available total dung in 1000 m^2 (=2 transects 250 m by 2 m wide)	Present sampling
	Cow dung	✓	g/100 m^2	Replicate (averaged to site/campaign)	Estimated available cow dung in 1000 m^2 (=2 transects 250 m by 2 m wide)	Present sampling
	Other dung	✓	g/100 m^2	Replicate (averaged to site/campaign)	Estimated available dung not from cow in 1000 m^2 (=2 transects 250 m by 2 m wide)	Present sampling
	Dung richness		g/100 m^2	Replicate (averaged to site/campaign)	Number of dung types (ie. cow, sheep/goat, donkey/horse, dromedar, carnivore, other)	Present sampling
	Cow presence		count	Replicate (averaged to site/campaign)	Binary variable accounting for presence/absence of cows	Present sampling
**Space**						
	Altitude over sea level	✓	m	Replicate (averaged to site)	Elevation over sea level	Hijmans et al. (2005)
	Moran eigenvector maps	✓	no units	Transect design	Several vectors of eigenvalues that describe spatial relations between sampling sites	Present sampling, Borcard et al. (2004)
**Climate**						
	Mean annual aridity	✓	no units	Replicate (averaged to site)	MAP / MAE (=Mean Annual Precipitation / Mean Annual potential Evapo-transpiration)	Trabucco & Zomer (2009)
	Mean monthly extraterrestrial radiation	✓	W/m^2	Replicate (averaged to site/season)	Mean monthly potential evapotranspiration (1950-2000) for each campaign (april-sept)	Trabucco & Zomer (2009)
	Mean monthly precipitation	✓	mm	Replicate (averaged to site/season)	Mean monthly precipitation (1950-2000) for each campaign (april-sept)	Hijmans et al. (2005)
	Mean annual precipitation	✓	mm	Replicate (averaged to site/year)	Mean annual precipitation (1950-2000) for each year (2013-2014)	Hijmans et al. (2005)
	Mean monthly temperature	✓	ºC	Replicate (averaged to site/season)	Mean monthly temperature (1950-2000) for each campaign (april-sept)	Hijmans et al. (2005)
	Mean site temperature	✓	ºC	Site/campaign	Mean temperature (averaged from values every 20min during 72h trap activity)	Present sampling
	Standard deviation site temperature	✓	ºC	Site/campaign	Standard deviation of temperatures measured every 20min during 72h trap activity	Present sampling
	Max site temperature		ºC	Site/campaign	Maximum temperature measured every 20min during 72h trap activity	Present sampling
	Min site temperature		ºC	Site/campaign	Minimum temperature measured every 20min during 72h trap activity	Present sampling
	Mode site temperature		ºC	Site/campaign	Mode of temperatures measured every 20min during 72h trap activity	Present sampling
	Max (percentile 80) site temperature		ºC	Site/campaign	Maximum (percentile 80) of temperatures measured every 20min during 72h trap activity	Present sampling
	Min (percentile 20) site temperature		ºC	Site/campaign	Minimum (percentile 20) of temperatures measured every 20min during 72h trap activity	Present sampling
	20-80% range site temperature		ºC	Site/campaign	Range (percentile 20 to percentile 80) of temperatures measured every 20min during 72h trap activity	Present sampling
	Percentage of 24h with temperature >10ºc		% day	Site/campaign	Percentage of day (24h) in which temperature >10ºc measured every 20min during 27h trap activity	Present sampling
	Percentage of 24h with temperature >15ºc		% day	Site/campaign	Percentage of day (24h) in which temperature >15ºc measured every 20min during 27h trap activity	Present sampling
	Percentage of 24h with temperature >20ºc		% day	Site/campaign	Percentage of day (24h) in which temperature >20ºc measured every 20min during 27h trap activity	Present sampling
	Percentage of 24h with temperature >25ºc		% day	Site/campaign	Percentage of day (24h) in which temperature >25ºc measured every 20min during 27h trap activity	Present sampling
	Mean monthly potential evapo-transpiration		mm	Replicate (averaged to site/season)	Mean monthly potential evapotranspiration (1950-2000) for each campaign (april-sept)	Trabucco & Zomer (2009)
**Soil**						
	Bare soil cover	✓	%	Replicate (averaged to site/campaign)	Visually estimated bare soil cover (no vegetation) over an area of ca. 1000 m^2	Present sampling, Tovar (2015)
	Soil structure 0-10 cm	✓	0,1,2	Replicate (averaged to site)	Visually estimated soil clustering (upper 0-10cm of 3cores, ø4cm)	Present sampling, Tovar (2015)
	Soil hardness 0-10 cm	✓	0,1,2	Replicate (averaged to site)	Manual estimation of soil resistance to compression (upper 0-10cm of 3cores, ø4cm)	Present sampling, Tovar (2015)
	Gravel content 0-10 cm	✓	%	Replicate (averaged to site)	Soil fraction of over 2mm (upper 0-10cm of 3cores, ø4cm)	Present sampling, Tovar (2015)
	Sand content 0-10 cm	✓	%	Replicate (averaged to site)	Soil fraction suspended in water after 3 min (Boyoucos method) (upper 0-10cm of 3cores, ø4cm)	Present sampling, Tovar (2015)
	Silt content 0-10 cm	✓	%	Replicate (averaged to site)	Soil fraction left after calculating sand and clay contents (upper 0-10cm of 3cores, ø4cm)	Present sampling, Tovar (2015)
	Clay content 0-10 cm	✓	%	Replicate (averaged to site)	Soil fraction suspended in water after 90 min (Boyoucos method) (upper 0-10cm of 3cores, ø4cm)	Present sampling, Tovar (2015)
	Bulk density 0-10 cm	✓	g/cm^3	Replicate (averaged to site)	Dry mass / volume (upper 0-10cm of 3cores, ø4cm)	Present sampling, Tovar (2015)
	Water field capacity 0-10 cm	✓	m^3 / m^3	Replicate (averaged to site)	Water content extracted from soil at 33 kPa (Richards Membrane Method) (upper 0-10cm of 3cores, ø4cm)	Present sampling, Tovar (2015)
	Permanent wilting point 0-10 cm		m^3 / m^3	Replicate (averaged to site)	Water content extracted from soil at 1500 kPa (Richards Membrane Method) (upper 0-10cm of 3cores, ø4cm)	Present sampling, Tovar (2015)
	Available water 0-10 cm		m^3 / m^3	Replicate (averaged to site)	Water content retained by soil (mass difference after 24h at 105ºC) (upper 0-10cm of 3cores, ø4cm)	Present sampling, Tovar (2015)
	Soil structure 10-20 cm		0,1,2	Replicate (averaged to site)	Same as above, middle 10-20cm segment of 3 soil cores (ø4cm, 30cm deep)	Present sampling, Tovar (2015)
	Soil hardness 10-20 cm		0,1,2	Replicate (averaged to site)	Same as above, middle 10-20cm segment of 3 soil cores (ø4cm, 30cm deep)	Present sampling, Tovar (2015)
	Gravel content 10-20 cm		%	Replicate (averaged to site)	Same as above, middle 10-20cm segment of 3 soil cores (ø4cm, 30cm deep)	Present sampling, Tovar (2015)
	Sand content 10-20 cm		%	Replicate (averaged to site)	Same as above, middle 10-20cm segment of 3 soil cores (ø4cm, 30cm deep)	Present sampling, Tovar (2015)
	Silt content 10-20 cm		%	Replicate (averaged to site)	Same as above, middle 10-20cm segment of 3 soil cores (ø4cm, 30cm deep)	Present sampling, Tovar (2015)
	Clay content 10-20 cm		%	Replicate (averaged to site)	Same as above, middle 10-20cm segment of 3 soil cores (ø4cm, 30cm deep)	Present sampling, Tovar (2015)
	Bulk density 10-20 cm		g/cm^3	Replicate (averaged to site)	Same as above, middle 10-20cm segment of 3 soil cores (ø4cm, 30cm deep)	Present sampling, Tovar (2015)
	Water field capacity 10-20 cm		m^3 / m^3	Replicate (averaged to site)	Same as above, middle 10-20cm segment of 3 soil cores (ø4cm, 30cm deep)	Present sampling, Tovar (2015)
	Permanent wilting point 10-20 cm		m^3 / m^3	Replicate (averaged to site)	Same as above, middle 10-20cm segment of 3 soil cores (ø4cm, 30cm deep)	Present sampling, Tovar (2015)
	Available water 10-20 cm		m^3 / m^3	Replicate (averaged to site)	Same as above, middle 10-20cm segment of 3 soil cores (ø4cm, 30cm deep)	Present sampling, Tovar (2015)
	Soil structure 20-30 cm		0,1,2	Replicate (averaged to site)	Same as above, lower 20-30cm segment of 3 soil cores (ø4cm, 30cm deep)	Present sampling, Tovar (2015)
	Soil hardness 20-30 cm		0,1,2	Replicate (averaged to site)	Same as above, lower 20-30cm segment of 3 soil cores (ø4cm, 30cm deep)	Present sampling, Tovar (2015)
	Gravel content 20-30 cm		%	Replicate (averaged to site)	Same as above, lower 20-30cm segment of 3 soil cores (ø4cm, 30cm deep)	Present sampling, Tovar (2015)
	Sand content 20-30 cm		%	Replicate (averaged to site)	Same as above, lower 20-30cm segment of 3 soil cores (ø4cm, 30cm deep)	Present sampling, Tovar (2015)
	Silt content 20-30 cm		%	Replicate (averaged to site)	Same as above, lower 20-30cm segment of 3 soil cores (ø4cm, 30cm deep)	Present sampling, Tovar (2015)
	Clay content 20-30 cm		%	Replicate (averaged to site)	Same as above, lower 20-30cm segment of 3 soil cores (ø4cm, 30cm deep)	Present sampling, Tovar (2015)
	Bulk density 20-30 cm		g/cm^3	Replicate (averaged to site)	Same as above, lower 20-30cm segment of 3 soil cores (ø4cm, 30cm deep)	Present sampling, Tovar (2015)
	Water field capacity 20-30 cm		m^3 / m^3	Replicate (averaged to site)	Same as above, lower 20-30cm segment of 3 soil cores (ø4cm, 30cm deep)	Present sampling, Tovar (2015)
	Permanent wilting point 20-30 cm		m^3 / m^3	Replicate (averaged to site)	Same as above, lower 20-30cm segment of 3 soil cores (ø4cm, 30cm deep)	Present sampling, Tovar (2015)
	Available water 20-30 cm		m^3 / m^3	Replicate (averaged to site)	Same as above, lower 20-30cm segment of 3 soil cores (ø4cm, 30cm deep)	Present sampling, Tovar (2015)

Supplementary S1, Table 2.

Site	Municipality	Replicate	Latitude	Longitude	Aridity	Altitude	Cow dung (gr/100m²)	Other dung (gr/100m²)
mor10	Saïdia	a	35.08605 N	2.28714 W	2864	1	1291.61	1385.54
mor10		b	35.08260 N	2.27282 W	2871	5	709.71	1195.24
mor9	Bni-Drar	a	34.89106 N	2.01928 W	2694	467	737.75	1299.58
mor9		b	34.88659 N	2.00943 W	2671	453	590.67	1238.22
mor8	Guenfouda	a	34.45488 N	2.03989 W	2476	842	0	1106.00
mor8		b	34.44317 N	2.03771 W	2443	852	16.25	1103.75
mor7	North of Aïn-Beni Mathar	a	34.14451 N	2.05680 W	2278	907	0	1061.67
mor7		b	34.13533 N	2.05412 W	2278	906	0	1061.33
mor6	South of Aïn-Beni Mathar	a	33.78164 N	1.99615 W	2228	1078	0	1102.00
mor6		b	33.79516 N	1.99353 W	2236	1076	0	1104.00
mor5	North of Tendrara	a	33.26954 N	1.98332 W	1934	1223	35.00	1064.00
mor5		b	33.28416 N	1.97771 W	1952	1213	0	1055.00
mor4	Between Tendrara and Bouarfa	a	32.83676 N	2.06539 W	1714	1329	17.75	1020.25
mor4		b	32.85788 N	2.06149 W	1755	1347	2.50	1034.83
mor3	East of Bouarfa	a	32.50570 N	1.89055 W	1206	1127	0	777.67
mor3		b	32.21061 N	1.68693 W	1213	1319	0	844.00
mor2	Between Bouarfa and Figuig	a	32.20064 N	1.66292 W	1170	1283	0	817.67
mor2		b	32.49803 N	1.87541 W	1214	1126	15.00	785.00
mor1	West of Figuig	a	32.14916 N	1.26801 W	894	905	0	599.67
mor1		b	32.15368 N	1.30344 W	900	897	0	599.00
