# Supplementary material for: Spatial and temporal variations of aridity shape dung beetle assemblages towards the Sahara desert
Source: PeerJ. 2018 Sep 20;6:e5210. doi: 10.7717/peerj.5210 (PMC6151256; doi:10.7717/peerj.5210)

Table 4. Species with significant Indicator Values (IndVal, DeCaceres & Legendre, 2009) per year (2013 and 2014) and season (wet and dry) at 10 sampling sites (spaced ca. 40 Km) along a ca. 400 Km transect from the Sahara desert to the semiarid Mediterranean coast (see map in Figure 1). Species were selected for three sections of the gradient in the wet season and only for two sections for the dry season following the groupings of a cluster analysis based on Bray-Curtis dissimilarity index.

Wet Season

2013				2014			
Semiarid	stat	p.value	signif	Semiarid	stat	p.value	signif
Amidorus cribricollis	1	0.010	**	Aphodius foetidus	1	0.025	*
Euonthophagus crocatus	1	0.010	**	Euonthophagus crocatus	1	0.025	*
Onitis numida	1	0.010	**	Onitis numida	1	0.025	*
Onthophagus maki	1	0.010	**	Onthophagus maki	1	0.025	*
Onthophagus andalusicus	1	0.010	**	Onthophagus andalusicus	1	0.025	*
Onthophagus taurus	1	0.010	**	Onthophagus opacicollis	1	0.025	*
				Onthophagus taurus	0.972	0.02	*
				Calamosternus mayeri	0.863	0.035	*
Intermediate section				Intermediate section			
Mecynodes leucopterus	0.853	0.030	*	Alocoderus hydrochaeris	1	0.005	**
Onthophagus nebulosus	0.698	0.025	*	Mecynodes leucopterus	0.859	0.005	**
				Onthophagus nebulosus	0.733	0.01	**
Desert				Desert			
Bodilus beduinus	0.918	0.005	**	Bodilus beduinus	0.887	0.025	*
Chilothorax hieroglyphicus	0.897	0.005	**	Chilothorax hieroglyphicus	0.835	0.045	*

Dry Season

2013				2014			
Semiarid	stat	p.value	signif	Semiarid	stat	p.value	signif
Anomius baeticus	0.983	0.015	*	Anomius baeticus	0.99	0.025	*
Rest of gradient				Rest of gradient			
Chilothorax hieroglyphicus	1	0.015	*	Bodilus beduinus	0.956	0.025	*
Bodilus beduinus	0.98	0.015	*				

Table 5. Summary of the GLRs evaluating the effects of the extracted PLS components on species richness and log abundance for the wet and dry seasons. Values in bold indicate regression coefficients of components that differ significantly from zero after Bonferroni correction ($p < 0.0125$).

	Estimate ± SE	t	p
Richness			
Wet season			
**Intercept**	**18.000 ± 0.316**	**56.873**	**< 0.0001**
**Component 1**	**1.070 ± 0.087**	**12.280**	**< 0.0001**
**Component 2**	**1.105 ± 0.213**	**5.186**	**0.0020**
**Component 3**	**0.850 ± 0.213**	**3.995**	**0.0072**
Dry season			
**Intercept**	**8.600 ± 0.523**	**16.452**	**< 0.0001**
Component 1	0.259 ± 0.168	1.547	0.1600
Abundance			
Wet season			
**Intercept**	**1.742 ± 0.116**	**14.964**	**< 0.0001**
Component 1	0.143 ± 0.053	2.707	0.0268
Dry season			
**Intercept**	**2.065 ± 0.125**	**16.495**	**< 0.0001**
**Component 1**	**0.135 ± 0.037**	**3.692**	**0.0061**

Figure 1. Variation partitioning of Dung Beetle community variance in raw species composition (based on RDA), beta diversity and Bray-Curtis dissimilarities (based on dbRDA) along the studied aridity gradient. Community variations in four sampling campaigns: two consecutive years in the wet season (after the rainy season) and two consecutive years in the dry season (before the rainy season) are explained by up to four groups of variables (climate, space, soil and dung availability). Numbers in each fraction are adjusted R^2 values. Statistical significance of testable pure fractions (based on partial RDA or dbRDA) are shown with * ($p < 0.05$) and ** ($p < 0.01$).

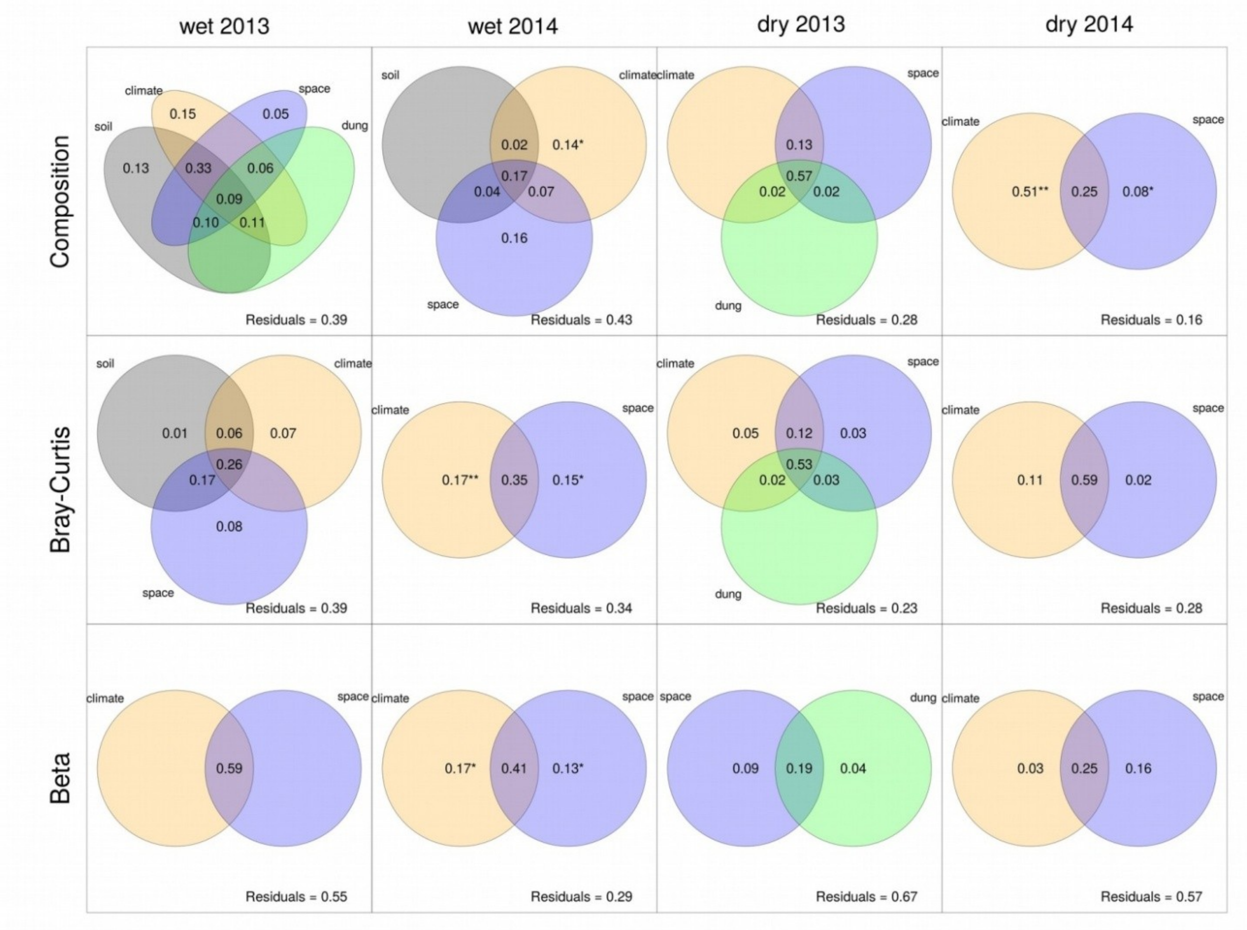

Supplement: Supplemental Information 3 — Table S4. Species with significant Indicator Values (IndVal, De Caceres & Legendre, 2009) per year (2013 and 2014) and season (wet and dry). Table S5. Summary of the GLRs evaluating the effects of the extracted PLS components on species richness and log abundance for the wet and dry seasons. Figure S1. Variation partitioning of Dung Beetle community variance in raw species composition (based on RDA), beta diversity and Bray-Curtis dissimilarities (based on dbRDA) along the studied aridity gradient. [file peerj-06-5210-s003.pdf]
